# Deep learning for sensitive detection of *Helicobacter Pylori* in gastric biopsies

**DOI:** 10.1186/s12876-020-01494-7

**Published:** 2020-12-11

**Authors:** Sebastian Klein, Jacob Gildenblat, Michaele Angelika Ihle, Sabine Merkelbach-Bruse, Ka-Won Noh, Martin Peifer, Alexander Quaas, Reinhard Büttner

**Affiliations:** 1 Else-Kröner-Forschungskolleg, Clonal Evolution in Cancer, University Hospital Cologne, Cologne, Germany; 2 Institute for Pathology, University Hospital Cologne, Cologne, Germany; 3 DeePathology.ai, Raanana, Israel; 4 Department of Translational Genomics, Center of Integrated Oncology Cologne-Bonn, Medical Faculty, University of Cologne, 50931 Cologne, Germany

**Keywords:** Artificial intelligence, Deep learning, Convolutional neural networks, Gastric cancer prevention, Screening, *Helicobacter pylori*

## Abstract

**Background:**

*Helicobacter pylori*, a 2 × 1 μm spiral-shaped bacterium, is the most common risk factor for gastric cancer worldwide. Clinically, patients presenting with symptoms of gastritis, routinely undergo gastric biopsies. The following histo-morphological evaluation dictates therapeutic decisions, where antibiotics are used for *H. pylori* eradication. There is a strong rational to accelerate the detection process of *H. pylori* on histological specimens, using novel technologies, such as deep learning.

**Methods:**

We designed a deep-learning-based decision support algorithm that can be applied on regular whole slide images of gastric biopsies. In detail, we can detect *H. pylori* both on Giemsa- and regular H&E stained whole slide images.

**Results:**

With the help of our decision support algorithm, we show an increased sensitivity in a subset of 87 cases that underwent additional PCR- and immunohistochemical testing to define a sensitive ground truth of HP presence. For Giemsa stained sections, the decision support algorithm achieved a sensitivity of 100% compared to 68.4% (microscopic diagnosis), with a tolerable specificity of 66.2% for the decision support algorithm compared to 92.6 (microscopic diagnosis).

**Conclusion:**

Together, we provide the first evidence of a decision support algorithm proving as a sensitive screening option for *H. pylori* that can potentially aid pathologists to accurately diagnose *H. pylori* presence on gastric biopsies.

## Background

*Helicobacter pylori* is a gram-negative bacterium, measuring 2–4 μm in length and 1 μm in width, usually presented in a spiral-shaped structure [1–3]. Clinically, *H. pylori* has been classified as a WHO class 1 carcinogen and represents the most common cause of gastric cancer worldwide [4, 5]. Importantly, the vast majority of all gastric cancers outside the cardia arise within *H. pylori* infected gastric mucosa [6, 7]. It has been shown that eradication of *H. pylori* can reduce the risk of gastric cancer in both retrospective-, as well as prospective clinical trials [8–10]. Although the incidence of gastric cancer itself is declining in Western countries, the disease is usually diagnosed in late stages, where individuals face a dismal prognosis—due to limited therapeutic options [11–13]. Meanwhile, systematic testing for *H. pylori* and corresponding therapeutic interventions have been established [14, 15]. However, the prevalence of *H. pylori* infection differs greatly between 20 and 80% within populations [16].

Advances in the field of hardware components, as well as the availability of large amounts of data, have allowed the field of artificial intelligence to rapidly grow [17]. Lately, these technologies have been successfully applied to improve diagnostic procedures in the medical field [1–3].

Therefore, we aimed to (1) design a deep learning based decision support algorithm that highlights *H. pylori* bacteria in image regions of gastric biopsies samples that are routinely tested for *H. pylori* presence and (2) validate this algorithm both on Giemsa stains and regular H&E stains comparing with microscopic diagnosis, immunohistochemistry and PCR.

## Methods

### Digitalization of whole slide images and quality control

Modified Giemsa and regular H&E stained slides were scanned using a NanoZoomer S360 (Hamamatsu Photonics) whole-slide scanning device at 40X magnification, as well as a DP200 slide scanner (ROCHE Diagnostics) at 40X. The slides were then evaluated for image quality and included within the validation cohort, if at least 50% of the tissue allowed a distinction of cell types. The evaluated tissue had to include at least 50% of gastric tissue to be included. Therefore, biopsies that primarily included intestine or esophageal tissue were not included.

### Detection of *H. pylori*: image processing

**Fig. 1:**
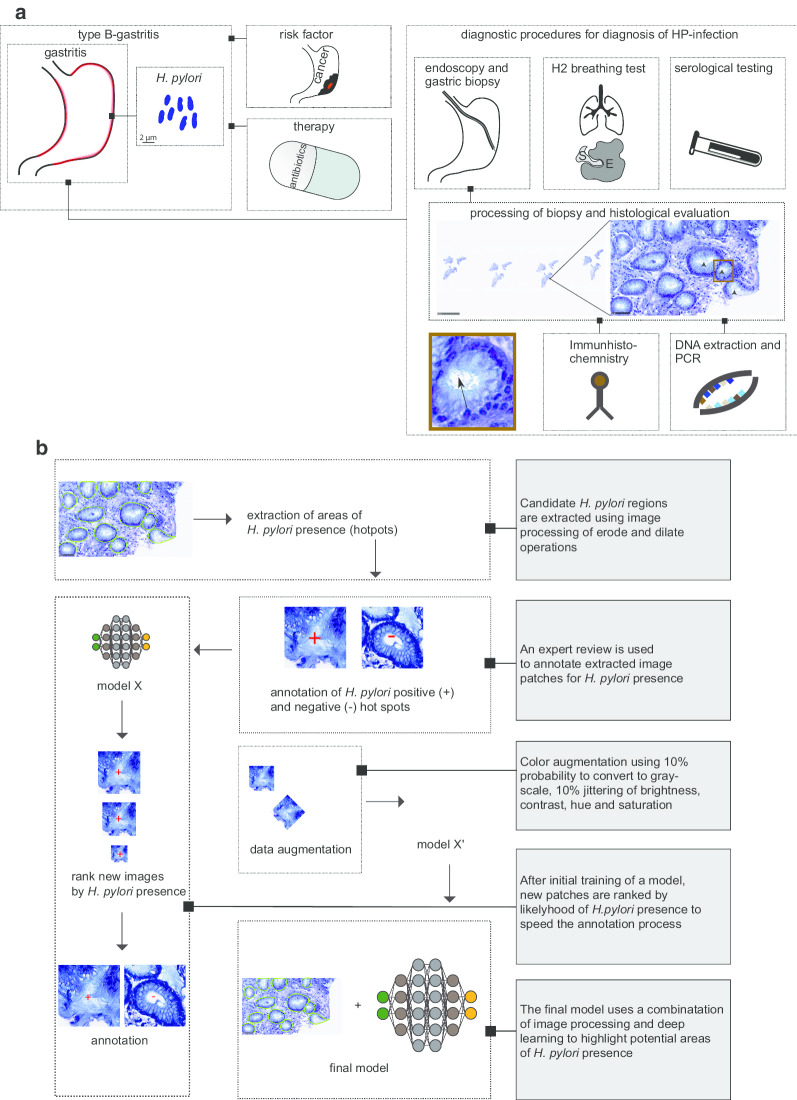
Clinical relevance of *H. pylori* and illustration of strategy to build a model detecting *H. pylori*. **a** Illustration of the diagnostic spectrum for type B-gastritis (bacterial gastritis, or *H. pylori* -gastritis) linked to *H. pylori* infection. While endoscopic evaluation of the stomach remains, and consequently harvesting gastric biopsies, other diagnostic tests, such as H2 breathing test and serological testing for *H. pylori* can be applied but may not differentiate for an active *H. pylori* infection. The tissue of gastric biopsies can histologically be reviewed, but also further tests can be applied, such as Immunohistochemistry (IHC) and Polymerase chain reaction (PCR), which are more sensitive. **b** Schematic representation of the approach to build a *H. pylori* classifier. Initially, areas within gastric biopsies of *H. pylori* presence were extracted (*H. pylori* hot spots, circled with green). Then, these hot spots were annotated according to their presence or absence of *H. pylori*, following a training step of an initial model. To further improve the detection sensitivity and specificity, this step was repeated for several times to generate a larger training dataset. The final model was trained containing several thousand *H. pylori* hot spots. Lastly, data was augmented by using color augmentation

Our approach consists of first localizing areas of *H. pylori* presence (herein referred as *H. pylori* hot spots) using image processing, and then cropping the hot spots into 224 × 224 patches and classifying them with a deep neural network. *H. pylori* typically exists inside and around glandular structures that can be described as white regions image regions inside the gastric tissue (Fig. 1b). To localize these regions, we downscaled the slide by a scale of × 32 in each axis, applied Otsu thresholding on the saturation channel in the HSV color space, then performed erode/dilate morphological operations to create a mask with the white regions. Then we would find the contours of these regions and crop them into non-overlapping patches of 224 × 224. Regions that had an area smaller than 32 pixels were discarded. A typical slide had hundreds to thousands of *H. pylori* patch candidates. These were then filtered by a classification network to only keep patches that contained *H. pylori*.

### Detection of *H. pylori*: deep learning

To classify the patches, we trained a compact VGG-style deep neural network [18]. The same network was trained on both Giemsa and H&E slides, in order to improve the generalization of the network on slides with stain variation. The network had 9 convolutional blocks followed by 3 fully connected layers. The first two convolutional layers are pointwise convolutions that help the network generalize on multiple stains [20]. The next two layers had kernel sizes of 7 × 7 and 5 × 5, that increased the receptive field, and the rest had kernel sizes of 3 × 3. The capacity of the network was reduced by scaling the number of features in every layer. The last convolutional layer had the output of only 32 channels. The first two fully connected layers had 1024 channels each, and the last layer has two outputs. We used 50% dropout between the fully connected layers, and batch normalization layers after the ReLU nonlinearity layers in the convolutional part of the network. In addition, we used standard cross entropy as the loss function and weighed the categories by their proportions in the dataset. As an optimizer, we used Adam with default parameters. We used a batch size of × 32, that was split among 4 1080ti GPUs, using the PyTorch framework. To help generalize among different stains, we used aggressive color augmentation. The brightness, contrast, saturation, and hue of every image was randomly jittered with strong portions. We also randomly flipped and rotated the image, then applied random grayscale and local elastic transformations.

### Visualization of the convolutional neural network output

Although the network performs classification, we applied a technique to help with the localization of individual *H. pylori* bodies. We first applied the SmoothGrad technique and averaged the gradients of the category score with respect to noisy input image pixels [21]. The gradient image is then passed to a ReLU gate, to keep only positive gradients. The gradient image was then used as a threshold, and input image pixels that had lower gradients were masked out, in order to only retain pixels that were important for the network decision. The threshold was then set to the 99.8% gradient percentile to keep the top instances. Then, the gradient image was dilated, and we drew contours over connected components to highlight areas that had *H. pylori* bodies with high confidence.

### Training datasets and annotation strategy

An overview of the training dataset can be found in Table 1. Overall, 191 H&E and 286 Giemsa stained slides were used, with a total of 2629 tiles containing for Giemsa 790 and H&E. About 4241 and 1533 tiles without *H. pylori* -like bacterial structures were used for Giemsa and H&E, respectively.

**Table 1:** Corresponding anatomical sites of gastric biopsies being evaluated for both training and validation of the algorithm

Localisation	Training		Validation	
	Giemsa	H&E	Giemsa	H&E
Antrum	226	145	59	52
Corpus	35	30	17	11
Duondenum	15	2	6	5
Cardia	8	12	4	3
Fundus	2	2	1	0

Using the strategy of training an initial model to classify *H. pylori* hot spots (Fig. 1b), we annotated the whole slide image (WSI), avoiding the need to manually annotate *H. pylori* regions in slides. First, the *H. pylori* candidate localization algorithm was applied on every slide, creating thousands of crops on average. Crops from slides lacking *H. pylori* were automatically labeled as “not *H. pylori*.” Crops from slides containing *H. pylori* were labeled in a gallery by pathologists. Since the number of *H. pylori* may be scarce even in slides that do contain *H. pylori*, there was a high level of variance between categories that made the annotation challenging. We used an active learning approach, where the dataset was constructed in steps of labeling few hundred crops each time, then a model was trained, and the remaining unlabeled crops were ordered from high to low according to their *H. pylori* category score. In the next step, the labeling was done on high scoring *H. pylori* crops, dramatically increasing the rate of encountered *H. pylori* crops and making the dataset labeling time efficient. This process was stopped when the rate of appearing *H. pylori* crops was very low, after a few thousand labeled images. The dataset contains more Giemsa slides than H&E slides. Using active learning, we were able to reduce the reviewed crops from nearly a hundred thousand crops (the total number of extracted candidates) to several thousand.

### Validation: region of interest review

**Fig. 2:**
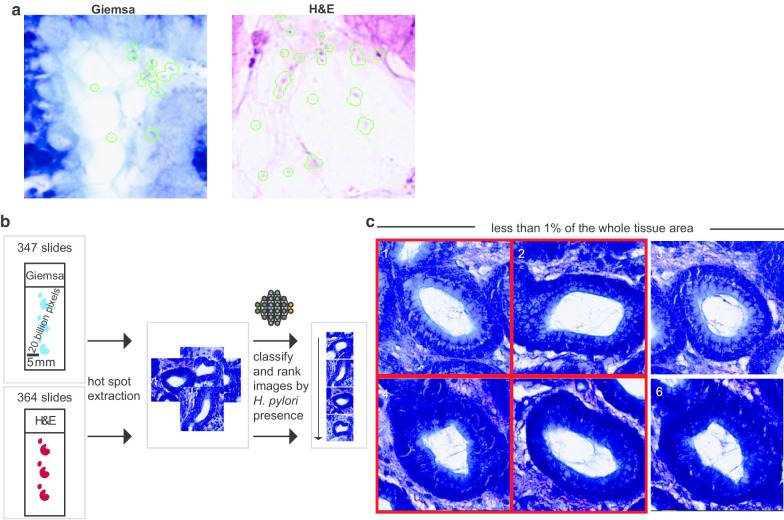
Validation strategy of the decision support algorithm. **a** Visualization of *H. pylori* detection in both Giemsa and H&E stained images. The green border highlights detected *H. pylori* bodies that the network correctly classified as *H. pylori*. **b** Illustration of the approach of validating the AI algorithm. About 347 Giemsa-stained slides (blue dots on illustrated slide) and 364 H&E slides (red dots on illustrated slide) were used, following an extraction of *H. pylori* hot spots. Then, the extracted hot spots were classified and ranked by *H. pylori* presence. The bean like structure of *H. pylori* is shown to visualize the scale. **c** The extracted and classified *H. pylori* hot spots were then annotated for *H. pylori* presence, as shown by a red box around four of the six tiles. The numbers correspond to an exemplified rank for *H. pylori* detection. For simplification, only Giemsa pictures are shown, while H&E stains were used for validation as well

We used a decision support algorithm approach of showing the top ranked 39 tiles (224 × 224 pixels) of an WSI to an experienced pathologist. We defined *H. pylori* presence, if at least two bacterial-like and spiral structures were present within an image. In total, the area of the presented fields reflected less than 1% of the area of the whole slide tissue area (Fig. 2b, c).

### Validation: RT-PCR and immunohistochemistry for *H. pylori*

In a subset of samples with conflicting microscopic diagnosis, despite clear visible *H. pylori*-like structures, or inconclusive *H. pylori* status, additional 46 samples were analyzed using PCR. *H. pylori* was screened with the RIDA GENE® Helicobacter pylori assay (r-biopharm, Darmstadt, Germany). At least one area on a hematoxylin–eosin stained slide was selected by an experienced pathologist (RB, AQ, SK) and DNA was extracted from corresponding unstained 10 µm thick slides of formalin-fixed, paraffin-embedded tissue by manual micro-dissection. Isolation was performed semi-automatically using the Maxwell® 16 FFPE Plus Tissue LEV DNA Purification Kit on the Maxwell® 16 System (Promega, Mannheim, GER) following the manufacturer’s protocols. The RIDA GENE® Helicobacter pylori assay was performed with 5 µl of DNA for each sample independent of DNA concentration and fragmentation on a CFX96 Touch™ Real-Time PCR Detection System (BIO-RAD, Hercules, California) according to the manufacturer’s protocol. Analyses were performed with the corresponding software. As amplification control, an internal control DNA (1 µl) was added to each PCR-Mix of all samples. All samples had to show a positive amplification of the internal control DNA (Additional file 1: Figure S2A). For a reliable detection of *H. pylori* and the Clarithromycin resistance positive (red curve) and negative controls (black curves) were included into each run (Fig. 2b). Additional file 1: Figure S2C shows the amplification curves for the detection of the clarithromycin resistance. The fluorescence signal for a resistance to clarithromycin had to be more than 20% of the fluorescence signal of the positive control. To determine the sensitivity of this assay, a serial dilution (1:2) of the positive control was performed (Additional file 1: Figure S2D). Discrepant cases were validated with the GenoTypeHelicoDR assay (Hain Lifesciences GmbH, Nehren, Germany) according to the manufacturer’s protocol with 5 µl of DNA for each sample. Immunohistochemical staining for *H. pylori* was performed on full tissue sections using the BOND MAX from Leica (Leica, Germany) according to the manufacturer’s protocol, and a polyclonal anti-*H. pylori* antibody from Cell Marque™. All stained sections were evaluated by an experienced pathologist (AQ, RB).

## Results

### Generating an assistive support algorithm to detect *Helicobacter pylori* on whole slide images

Symptoms of gastritis regularly require an endoscopic evaluation of the stomach. While several diagnostic tests can be applied, histo-morphological assessment of the tissue can be considered a standard procedure in Western countries (Fig. 1a) [4–10]. Clinically, the subtype of bacterial-associated gastritis (Type-B-Gastritis) is linked to infection with *H. pylori*. Usually, modified stains, such as Giemsa stains, help visualize these bacterial structures for histo-morphological assessment. In addition, H&E stains are performed to evaluate morphological abnormalities.

Therefore, we generated a decision support algorithm that would highlight regions of *H. pylori* presence on both modified Giemsa stains and regular H&E stains. We applied a combination of image processing and deep learning to extract regions of *H. pylori* presence, as well as to classify those regions, using a convolutional neural network (CNN) (Fig. 1b). Due to the fact this hybrid approach limits the amount of image information that are processed by a CNN-thereby saving computational resources–it allows the WSI analysis in high resolution to be done within seconds on regular clients. Typically, *H. pylori* resides in areas that can be described as a white background from an image processing point of view. Thus, we applied a pre-processing step to detect these candidate regions, using a combination of thresholding- and morphological operations on a low-resolution image. We decided to extract all candidate regions and annotate those patches in a gallery as the annotation of *H. pylori* containing areas is necessary, but time consuming and challenging on WSI (Fig. 1b). We applied an active learning approach on the annotated data, which represents an imbalanced data pool. The images were sorted by their *H. pylori* scores, allowing high-scoring images to be presented in priority.

In order to overcome the stain variation of histological specimens, we applied aggressive color augmentation on the extracted images for better performance and generalizability. Images were converted to a grayscale with 10% probability and heavy color jittering. In addition, we trained the same network for both H&E and Giemsa stains to further improve generalization across color- and stain variations.

Having generated the final model, we further aimed to understand the decisions of the network. We visualized and highlighted areas in the image that were significant for the network’s output (Fig. 2a). Having shown that the algorithm detects *H. pylori* bacterial structures on both modified Giemsa stains and regular H&E stains, we validated the algorithm using an independent cohort of gastric biopsies of cases that had not been used for training purpose. Initially, we validated the decision support technology against microscopic diagnosis.

### Comparison of *Helicobacter pylori* detection to microscopic diagnosis

#### Validation strategy

*H. pylori* is a small particle of a size of about 2–4 μm, which accounts for one pixel in an image containing about 20 billion pixels in total (Fig. 2b). For the purpose of validation, we applied a selective presentation of tiles that had been extracted and classified by the algorithm, showing less than 1% of the tissue of the whole slide in total. Finally, two board-certified pathologists (expert review) evaluated these tiles for the presence of *H. pylori* (Fig. 2c). No other information was shown to the pathologists reviewing the tiles for validation.

**Fig. 3:**
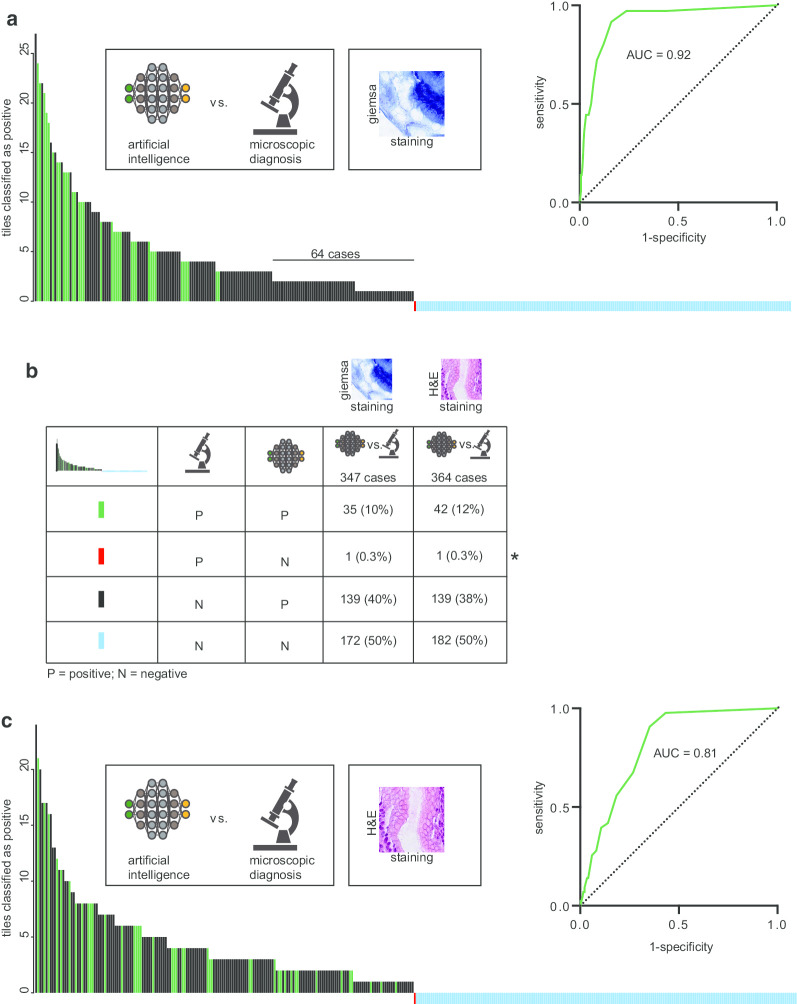
Validation of the decision support algorithm against microscopic diagnosis. **a** Bar chart of detection of *H. pylori* with help of the assistive algorithm, validated against microscopic diagnosis using Giemsa stains. The Y-axis reflects the amounts of detected positive tiles using the algorithm assisted approach. The color code of each bar is shown in legend of (**b**). **c** Calculation of area-under-the-curve (AUC) of *H. pylori* detection for H&E stains validated against microscopic diagnosis

For modified Giemsa stains, 347 slides were analyzed and compared to microscopic diagnosis (Fig. 3a, b). We defined *H. pylori* positivity as presence of at least two *H. pylori*-like bacterial structures within an extracted image. By using this definition, we detected *H. pylori* in 181 slides, with varying amounts of tiles being positive (Fig. 2a). Based on the amounts of positive tiles, the calculated area-under-the-curve (AUC) was 0.92 for modified Giemsa stains compared against microscopic diagnosis (Fig. 2a). To further provide evidence of the generalizability of the algorithm, we validated the model on regular H&E stains. For this purpose, 364 cases were used and validated against microscopic diagnosis. The AUC for H&E was 0.81 (Fig. 3c). Interestingly, a potential threshold of 2 tiles appeared to increase specificity without decreasing the sensitivity on Giemsa stains (Fig. 3a, Table 2), while this was not the case for H&E stains. Within H&E stains, there were cases found to be positive presenting with only one positive tile (Fig. 3c). In addition, there was one case that was found to be positive with help of histo-morphological diagnosis, but where the decision support algorithm could not reveal tiles containing *H. pylori* (Fig. 3b, refer to *). In further validation, it was confirmed that this case was *H. pylori* negative.

**Table 2:** Summary of the results of the validation, in respect to staining and validation techniques

Parameter (of 87 cases)	Microscope (Giemsa)	Algorithm (Giemsa)	Algorithm (tile based threshold > 2)	Ground truth (IHC/PCR)
True positive (n)	13	19	19	19
False positive (n)	5	36	23	NA
False negative (n)	6	0	0	NA
True negative (n)	63	32	45	68
Sensitivity (%)	68.4	100	100	NA
Specificity (%)	92.6	47.1	66.2	NA
Disease prevalence (%)	NA	NA	NA	21.8

*NA* not applicable

#### Validation of microscopic diagnosis against IHC/PCR

**Fig. 4:**
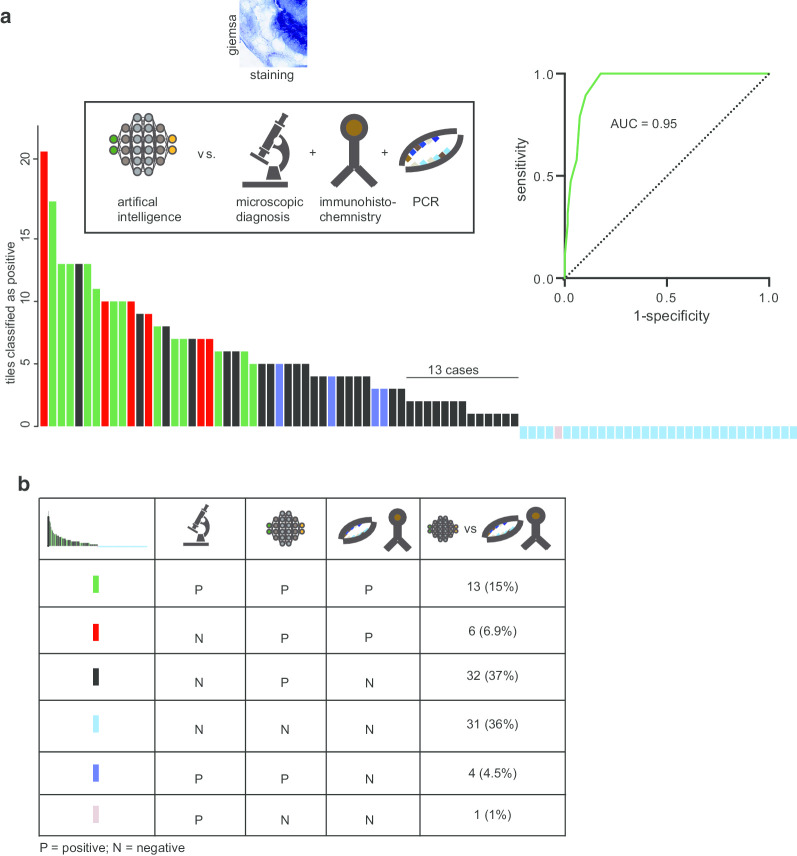
Validation of the decision support algorithm against microscopic diagnosis and IHC/PCR. **a** Bar chart of detection of *H. pylori* with help of the decision support algorithm, validated against microscopic diagnosis, and IHC/PCR using Giemsa stains. The Y-axis reflects the amounts of detected positive tiles using the algorithm. All 32 cases where the assistive approach did not detect *H. pylori*, underwent an additional PCR testing. The color code of each bar is shown in legend of (**b**). **c** Calculation of area-under-the-curve (AUC) of *H. pylori* detection for H&E stains validated against microscopic diagnosis

Having shown an initial performance of the algorithm, we applied two more sensitive and independent technologies to allow an additional validation (ground truth) of *H. pylori* presence on a subset of the cohort that had been initially validated against microscopic diagnosis. Cases with clear and visible *H. pylori* bodies in Immunohistochemistry (IHC) did not undergo additional PCR testing, while cases uncertain of *H. pylori* status after IHC, underwent additional PCR testing. 19 cases were confirmed to be *H. pylori* positive using IHC/PCR in this cohort and only 13 cases were identified as positive microscopically (Fig. 4a, b; Table 2). In addition to the 13 positive cases, 5 cases were identified microscopically as *H. pylori* positive, but these cases could not be confirmed by IHC/PCR (Fig. 4b, Table 2). Overall, microscopic diagnosis revealed a sensitivity of 68.4% with a specificity of 92%.

#### Validation of the decision support algorithm against IHC or PCR

Within the set of modified Giemsa stains, a subset of 87 slides was analyzed with the decision support algorithm. Out of 19 *H. pylori* positive cases that were confirmed by IHC/PCR, assisted approach was able to detect all 19 of them (Fig. 4a, b). Interestingly, the two microscopical positive cases that were detected negative by AI in the initial validation stage using microscopic diagnosis as ground truth were found to be negative by IHC and PCR testing (Fig. 4a, b). Together, an AUC of 0.95 was achieved for the assistive algorithm using Giemsa stains (Fig. 4a), while the sensitivity was 100% and the specificity 47.1% without applying a tile bases threshold (Table 2). Given that the initial validation against microscopic diagnosis showed 64 cases with 2 positive tiles that were found to be negative by microscopic assessment, this could be confirmed by further validating a subset of those cases against IHC/PCR. Indeed, 13 cases with two positive tiles were also negative, highlighting a potential threshold of more than 2 tiles to increase specificity (66.2%, Table 2), without decreasing the sensitivity (Fig. 4a, b).

## Discussion

Our approach follows the current regulations of the application of computerized algorithms, which require to visualize the results of a given technology. Those results can then be further evaluated by an expert and therefore be checked for plausibility. With the help of this assistive decision support algorithm, we avoided the black box character of AI. In detail, we did not assign a general category (positive/negative) on whole slide basis. Instead, by using a decision support algorithm that highlights regions of potential *H. pylori* presence, we were able to increase the sensitivity of *H. pylori* detection (microscope: 68.4%; decision support algorithm: 100%), and at the same time allowed it to be applied on both H&E and Giemsa stains, strengthening the idea of a more generalized approach.

During the course of this study, Martin et al. [22] published a slightly different approach by applying tissue segmentation of histological specimens of gastric biopsies to identify diseased mucosa. In this study, the gold standard was defined by methods other than PCR, including histological evaluation by a Pathologist. While this approach seems to be intriguing, one may not be able to diagnose cases with less characteristic histology but presence of *H. pylori* bacteria. In addition, our approach can be operated with limited computational resources because of its architecture and can be applied on both Giemsa and H&E stains.

In addition, several studies have applied deep learning for *H. pylori* detection on endoscopic imaging data [23–25]. Given that this intervention would not necessarily even require to harvest biopsies and therefore to collect tissue, it appears advantageous. However, within those studies more sensitive testing for the presence of *H. pylori*, including sequencing based *H. pylori* detection (PCR), would potentially have provided a definition of *H. pylori* status of higher sensitivity as a gold standard for evaluating the Computer-Aided-Detection (definition of ground truth). As chronic infection of the stomach displays an important risk factor for malignancies, including mucosa associated lymphoid tissue (MALT)-lymphoma and gastric cancer, one may argue that harvesting tissue for histological evaluation is a diagnostic necessity. Therefore, it remains to be seen whether endoscopic detection of *H. pylori* infection using deep learning would result in clinical benefit for individuals.

Technically, the architecture of our algorithm combines both image processing and classification by using a deep neural network. In summary, only relevant image areas (*H. pylori* -hotspots) are further analyzed by a deep neural network. This design of the algorithm greatly lowers the amount of computational resources that needs to be present to analyze whole slide images for *H. pylori* presence.

Both image quality and staining quality influence the specificity of the decision support algorithm. Structures, which were found to confuse the network, leading to detection of particles falsely classified as *H. pylori*, are shown in Additional file 2: Figure S1. Likely, this is due to either other bacterial structures being present on the slide—potentially contaminations of the tissue due to processing of the histological specimens—as well as image quality. Within this study, we included tissues that were processed using standard techniques. For potential clinical application of decision support algorithms to detect *H. pylori* on regular biopsies, a prior quality check of the specimens might allow to lower the detection rate of either detritus or image artifacts.

**Fig. 5:**
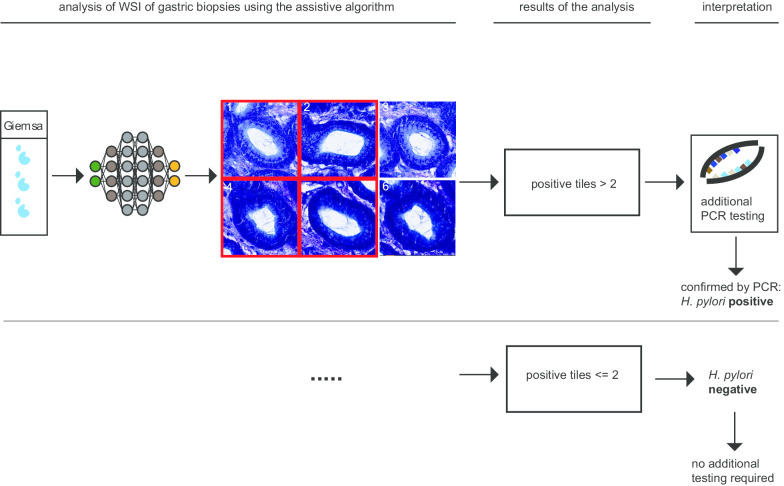
Potential workflow of a decision support algorithm to detect *H. pylori* on whole slide images. Processed images undergoing a visual confirmation of *H. pylori* presence (analysis, results) or either a definite diagnosis of *H. pylori* absence/presence (interpretation). Cases above a certain threshold (2 in our study) would undergo additional confirmation by PCR testing

We observed a prevalence of 22% of *H. pylori* infected individuals within the subset of IHC/PCR validated cases (Table 2). Considering the generalizability of the validation, the specificity might be lower compared to cohorts with a higher disease prevalence. Still, with a sensitivity of 100% for both H&E and specialized Giemsa stains, our approach could potentially be qualified for screening purpose. In addition, the performance of *H. pylori* detection using Giemsa stains was higher, potentially because the human interactor is trained to identify *H. pylori* bacterial structures on these stains. In light of the high false-positive rate (41%; 36 out of 87 cases) of the assistive algorithm applied on Giemsa stains (Fig. 4a, b; Table 2), a potential clinical application might require an additional validation step of PCR or IHC diagnosis, if a certain threshold of positive tiles been reached (Fig. 5, Table 2). At the same time, it allows to sensitively declare cases as *H. pylori* negative, if the decision support algorithm did not detect *H. pylori*. Potentially, this technology would therefore qualify as a sensitive screening technology. However, because experts will be involved in the decision making, more experience with digitalized images of *H. pylori* might further increase the specificity of these approaches. Furthermore, image quality and the quality of the stains greatly influence the ability of an observer to specifically recognize *H. pylori* -like bacteria structures.

## Conclusion

Our study highlights a discrepancy between microscopic diagnosis and *H. pylori* status, using sensitive diagnostic tools, such as IHC/PCR, which provides evidence of cases that are missed using microscopic diagnosis alone. This is in line with previous findings, where *H. pylori* DNA was found in about 30% of cases that had been diagnosed as *H. pylori* -negative, based on histo-morphological assessment [26]. Potentially, this further strengthens the idea to apply more sensitive screening options within the standard histo-morphological review process of pathologists.

## Supplementary information

**Additional file 1: Figure S2.** Validation and application of PCR to detect *H. pylori* on gastric biopsies. Representative results of the multiplex real-time PCR performed with the RIDA GENE^®^ Helicobacter pylori assay (r-biopharma, Darmstadt, Germany). (**A**) Amplification of the internal control DNA. (**B**) Amplification of the specific sequence for *H. pylori* (16SrRNA). Positive control is shown in red and the non-template control in black for each channel. (**C**) Amplification of the specific sequence for the detection of the Clarithromycin resistance (23S rRNA). Positive control is shown in red and the non-template control in black for each channel. (**D**) Serial dilution (1:2) of the positive control (5000 copies/μl starting concentration). The RIDA GENE^®^ Helicobacter pylori assay (r-biopharma, Darmstadt, Germany) can detect down to 9.8 copies/μl in an unknown sample. Shown are the fluorescence signals of the *H. pylori* channel (FAM/RFU). RFU: relative fluorescence units.

**Additional file 2: Figure S1.** Visualization of the decisions of the applied CNN and its false detections. (**A**–**D**) Synthetic images that maximize the *H. pylori* category score and the non- *H. pylori* category score. (**E**–**H**). Visualization results that confused the network, and which falsely lead to *H. pylori* detection (**I**). For visualization of the features the network searches for, we used the approach of Simonyan et al. [19]. A noise image is inserted to the network, a specific pixel and category in the network output is set as the target, and several iterations of gradient ascent are run in order to modify the input image pixels to receive a high value in the target pixel. Using this we created examples of input images, that caused a high activation at the target pixel for each of the categories. For creating smooth image visualizations, we followed the example of Smilkov et al. and used regularization by rotations, reflections, and normalization of the gradients. We observed that images maximizing the *H. pylori* category contained multiple *H. pylori* looking like bodies, and images maximizing the *H. pylori* category did not have these features.

## Data Availability

All data (whole slide images) used in the manuscript are available upon reasonable request to the corresponding author. DeePathology Ltd. will consider making the manuscript code available per request for academic institutions for non-clinical, academic research purposes.

## Abbreviations

AUC: Area under the curve
CNN: Convolutional neural network
*H. pylori*: *Helicobacter pylori*
WSI: Whole slide image
AI: Artificial intelligence
PCR: Polymerase chain reaction
