# Supplementary figures and images for: Deep learning for sensitive detection of Helicobacter Pylori in gastric biopsies
Source: BMC Gastroenterol. 2020 Dec 11;20:417. doi: 10.1186/s12876-020-01494-7 (PMC7731757; doi:10.1186/s12876-020-01494-7)

Figure S2

A

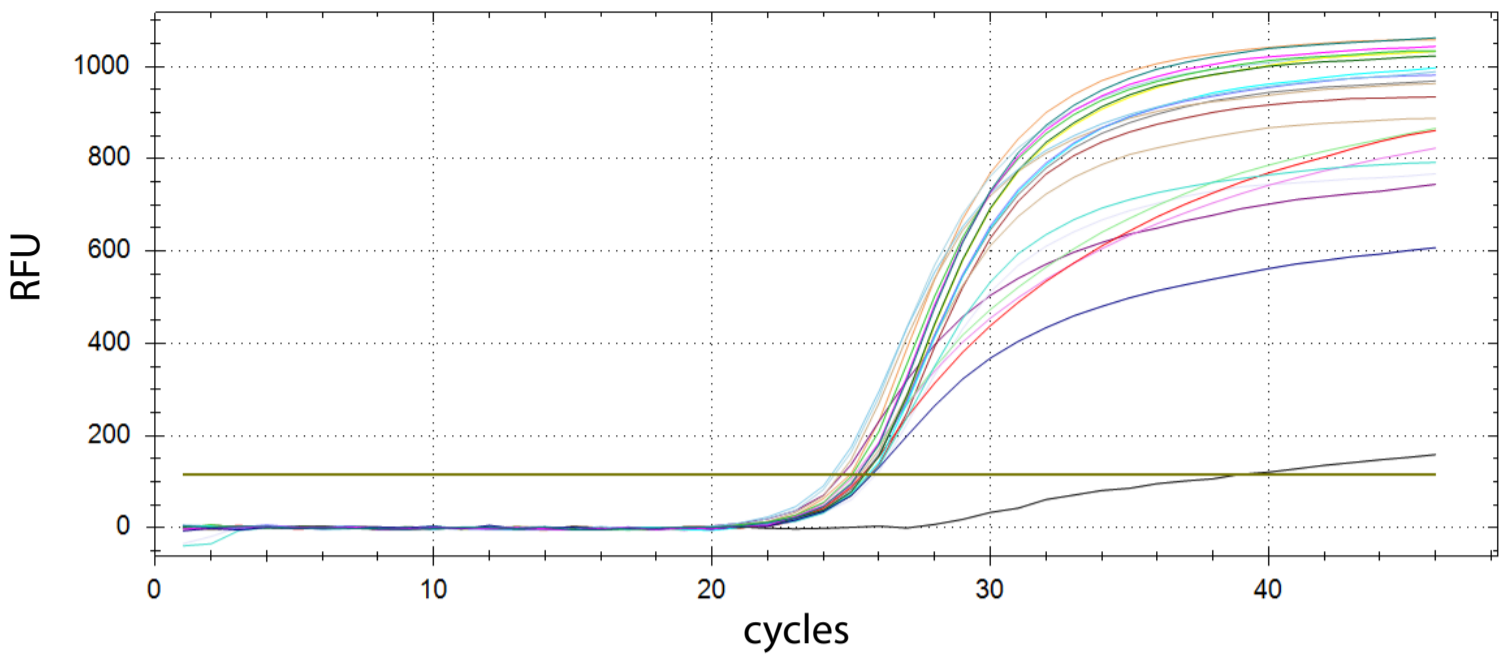

B

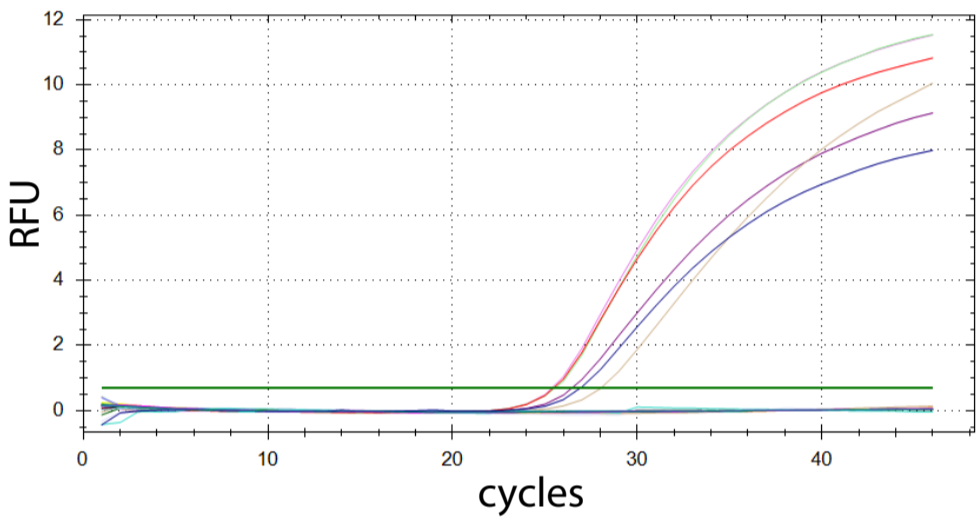

C

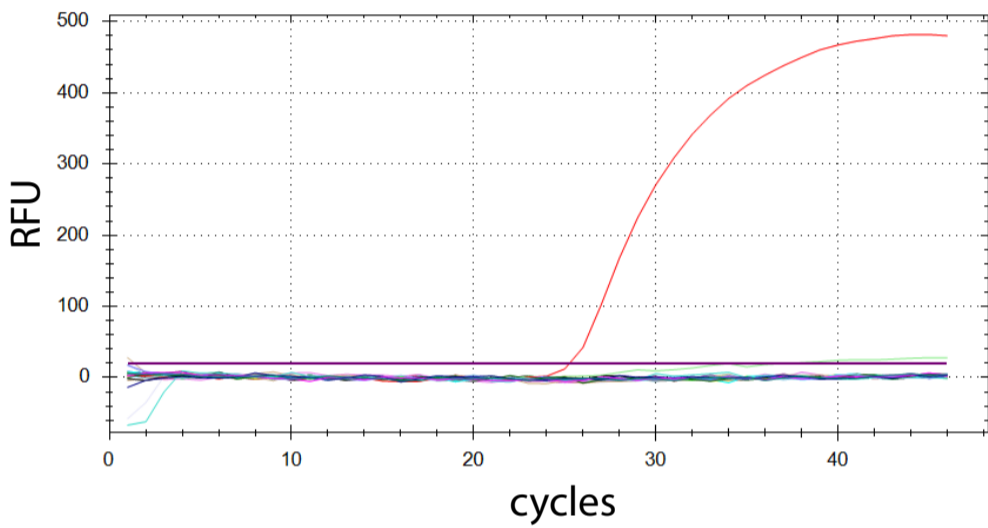

D

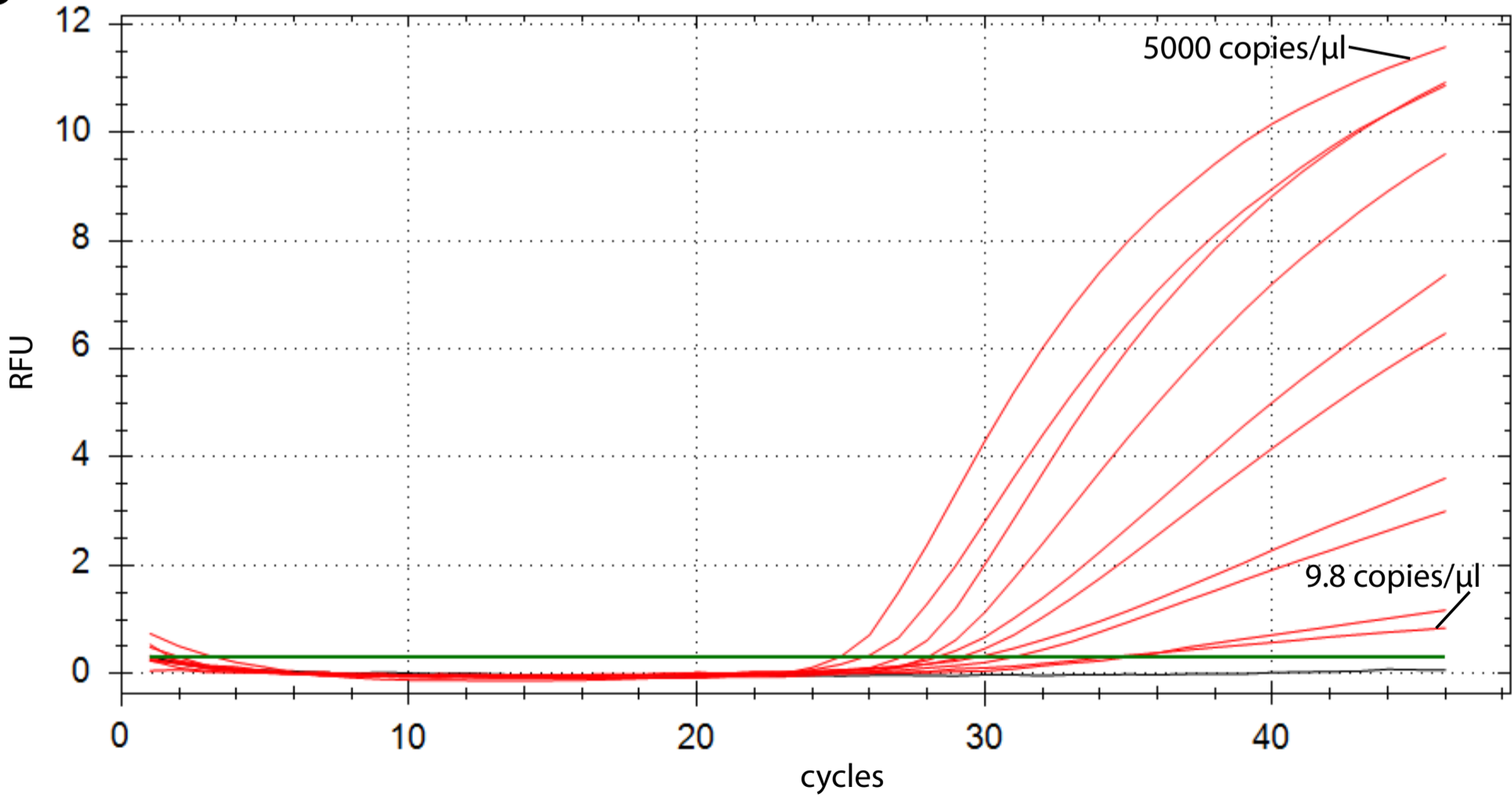

Supplement: Supplementary file 1 — Additional file 1: Figure S2. Validation and application of PCR to detect H. pylori on gastric biopsies. Representative results of the multiplex real-time PCR performed with the RIDA GENE® Helicobacter pylori assay (r-biopharma, Darmstadt, Germany). (A) Amplification of the internal control DNA. (B) Amplification of the specific sequence for H. pylori (16SrRNA). Positive control is shown in red and the non-template control in black for each channel. (C) Amplification of the specific sequence for the detection of the Clarithromycin resistance (23S rRNA). Positive control is shown in red and the non-template control in black for each channel. (D) Serial dilution (1:2) of the positive control (5000 copies/μl starting concentration). The RIDA GENE® Helicobacter pylori assay (r-biopharma, Darmstadt, Germany) can detect down to 9.8 copies/μl in an unknown sample. Shown are the fluorescence signals of the H. pylori channel (FAM/RFU). RFU: relative fluorescence units. [file 12876_2020_1494_MOESM1_ESM.pdf]

**Figure S1**

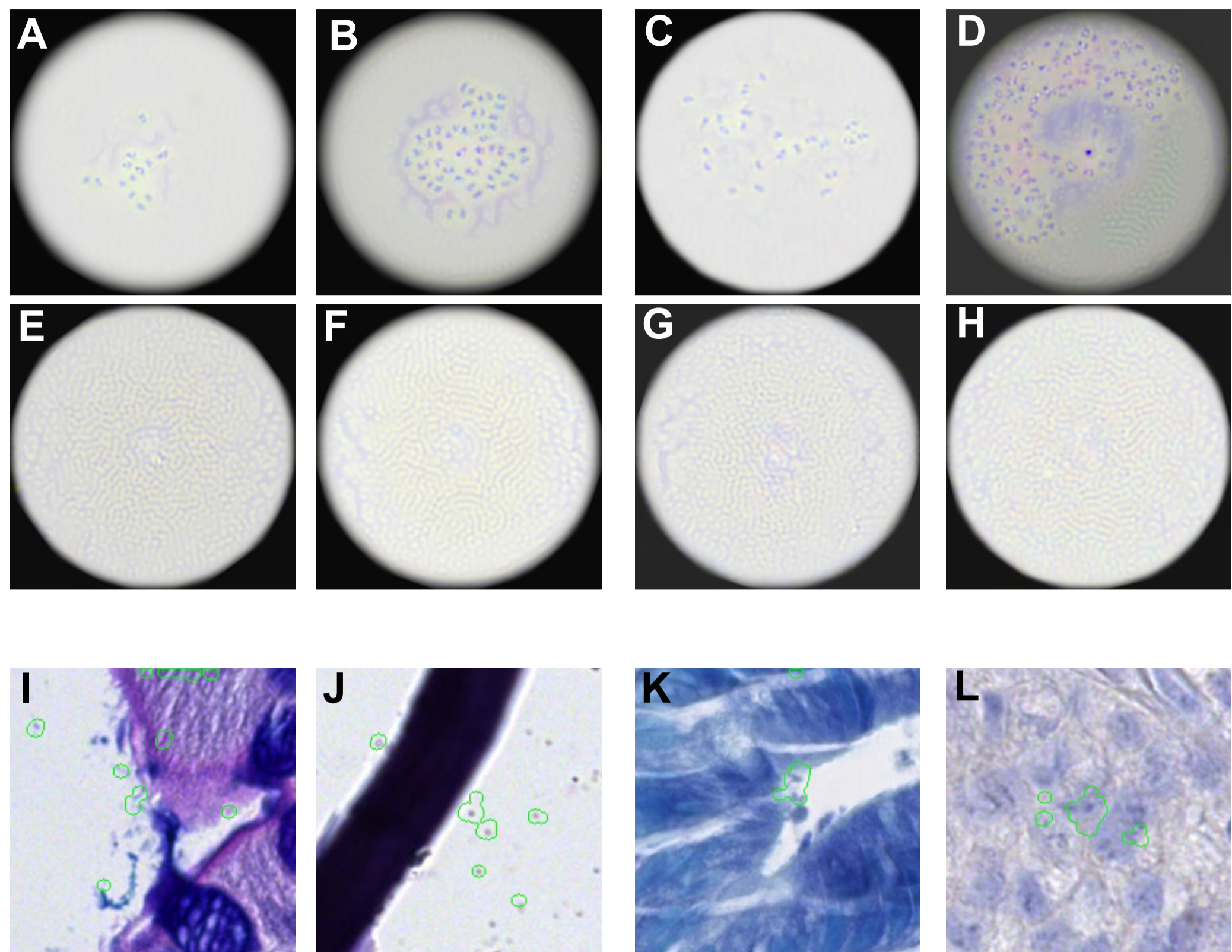

Supplement: Supplementary file 2 — Additional file 2: Figure S1. Visualization of the decisions of the applied CNN and its false detections. (A–D) Synthetic images that maximize the H. pylori category score and the non- H. pylori category score. (E–H). Visualization results that confused the network, and which falsely lead to H. pylori detection (I). For visualization of the features the network searches for, we used the approach of Simonyan et al. [19]. A noise image is inserted to the network, a specific pixel and category in the network output is set as the target, and several iterations of gradient ascent are run in order to modify the input image pixels to receive a high value in the target pixel. Using this we created examples of input images, that caused a high activation at the target pixel for each of the categories. For creating smooth image visualizations, we followed the example of Smilkov et al. and used regularization by rotations, reflections, and normalization of the gradients. We observed that images maximizing the H. pylori category contained multiple H. pylori looking like bodies, and images maximizing the H. pylori category did not have these features. [file 12876_2020_1494_MOESM2_ESM.pdf]
